# Visual outcomes and complications following one-way air-fluid exchange technique for vitreous hemorrhage post vitrectomy in proliferative diabetic retinopathy patients

**DOI:** 10.1186/s12886-021-01885-8

**Published:** 2021-03-09

**Authors:** Qun Wang, Jie Zhao, Qing Xu, Cui Han, Baojie Hou, Yifei Huang

**Affiliations:** 1 Ophthalmology Department, Third Medical Center of PLA General Hospital, No. 69, Yongding Road, Haidian District, Beijing, China; 2 Ophthalmology Department, First Medical Center of PLA General Hospital, No.29, Fuxing Road, Haidian District, Beijing, China

**Keywords:** Air-fluid exchange, Syringe, Vitreous hemorrhage, Par plana vitrectomy, Proliferative diabetic retinopathy

## Abstract

**Background:**

To evaluate the efficacy and outcomes of one-way surgical technique for the treatment of vitreous hemorrhage post vitrectomy on proliferative diabetic retinopathy (PDR) patients.

**Methods:**

This retrospective case series include 47 PDR patients who had vitrectomy with balanced saline solution tamponade and have developed vitreous hemorrhage without significant absorption. The one-way air-fluid exchange procedure which involves the application of a 0.22-μm pore size filter to exchange about 4.5–5.5 ml of fluid with a 10 ml syringe was performed on 47 patients (47 eyes). Post procedure, additional treatments were administered when needed. Best corrected visual acuity (BCVA), occurrence of intra-procedural and post-procedural complications were recorded and analyzed.

**Results:**

A total of 47 eyes of 47 PDR patients with a mean age of 50.8 ± 12.0 years were reviewed. Because of vitreous hemorrhage or tractional retinal detachment of PDR, all 47 eyes underwent vitrectomy with balanced saline solution tamponade prior to the exchange procedure. Four patients (8.51%) and 43 patients (91.5%) were diagnosed with type 1 diabetes mellitus (T1DM), or type 2 diabetes mellitus (T2DM), respectively. All 47 eyes were given the one-way air-fluid exchange procedure in the treatment room. Forty-two cases (89.4%) needed the air-fluid exchange procedure only once, 4 cases (8.51%) underwent the procedure twice, and 1 case (2.13%) was given the procedure three times, followed by additional retinal photocoagulation and one intravitreal injection of Conbercept. In addition to the procedure, no further treatment was needed for 5 eyes (10.6%) while additional retinal laser treatment was provided for 41 eyes (87.2%). The BCVA at the final follow-up was significantly improved from the initial acuity baseline in all cases. No complications were observed during the follow-ups.

**Conclusion:**

This one-way air-fluid exchange procedure can effectively exchange the vitreous hemorrhage and improve visual acuity of PDR patients who develop vitreous rehemorrhage post vitrectomy without obvious complications.

## Background

Vitreous cavity hemorrhage is a common problem post pars plana vitrectomy (PPV) for proliferative diabetic retinopathy. It has become a major concern for both surgeons and patients, even with recent advanced surgical techniques [1, 2]. Vitreous hemorrhage (VH) post PPV can affect the examniations of the fundus, detection of iatrogenic retinal breaks, feasibility of retinal photocoagulation and leave the patients with the risk for glaucoma [1, 3]. Postoperative VH also affects patients’ vision, and lead to the worries of the patients those whose visual acuity didn’t improved as expected, also, this may be especially crucial in monocular patients. Some of recurrent VH cleared up spontaneously within several weeks of their onset, while others required further surgery, to help the absorption of the hemorrhage [4, 5]. Vitreous cavity hemorrhage can be managed by either vitreous cavity lavage or air-fluid exchange procedures in-office [6–11]. Most papers that discussed the in-office air-fluid exchange usually focus on the technique or a modified technique and its application in a few clinical cases. We retrospectively reviewed the application of one-way air-fluid exchange technique at our hospital for 5 years and here report the visual outcomes and complications of the one-way air-fluid exchange procedure handling post-vitrectomy vitreous cavity hemorrhage using a syringe with a 0.22 μm pore size filter.

## Methods

This study identified the patients who had underwent vitrectomy and with balanced saline solution vitreous tamponade at the end of vitrectomy from December 1, 2014 to August 1, 2019 because of diabetes-related complications in our hospital. There were 47 patients who developed post-vitrectomy vitreous rehemorrhage and rebleeding lasting for 1 month without significant absorption. Data collected included baseline demographics, best-corrected visual acuity, indication for the air-fluid exchange, complication, outcome, and duration of follow-up. When these patients were retrospectively reviewed (Table 1), the VH was classified into three categories: either early postoperative (VH fewer than 4 weeks post-surgery but not present on postoperative day 1), delayed postoperative (VH 4 weeks or more post-surgery), and severe persistent (present since postoperative day 1 and nonclearing for more than 4 weeks) [12]. In order to confirm that there was no retinal detachment, prior to the air-fluid exchange procedure, an ultrasound scan was applicated to verify the severity of vitreous hemorrhage and the status of the retina. The single-needle vitreous cavity gas-liquid exchange procedure was then performed by the same surgeon in a treatment room. Written consent was obtained before the procedure from all 47 patients. Patients who had less than 6 months of follow-up were excluded. All the study procedures adhered to the recommendations of the Declaration of Helsinki. Ethic approval was obtained from Medical Ethics committee of Third medical center of PLA general hospital.

**Table 1:** Demographic and clinical characteristics of patients

Variables (mean ± SD,)	Total
Gender	
Male	27
Female	20
Age, years	50.8 ± 12.0
Indications for primary PPV	
VH	44
VH and TRD	3
Days of VH post PPV,	34.9 ± 42.7
Category of VH post PPV	
Early VH	33
Delayed VH	13
Severe persistent VH	1
Diagnosis	
T_1_DM	4
T_2_DM	43
Lens status	
Pseudophakic	21
Phakic	26
Times of Air-fluid exchange procedure	
= 1	42
= 2	4
= 3	1
Additional ocular treatment	
No treatment	5
Only photocoagulation	41
Laser plus Conbercept	1
Follow-up post last exchange, months	22.7 ± 13.6

*VH* Vitreous hemorrhage; *TRD* Tractional retinal detachment; *PPV* Pars plana vitrectomy; *DM* Diabetes mellitus. Conbercept: an anti-VEGF drug for intraocular use in China

### Surgical technique

**Fig. 1:**
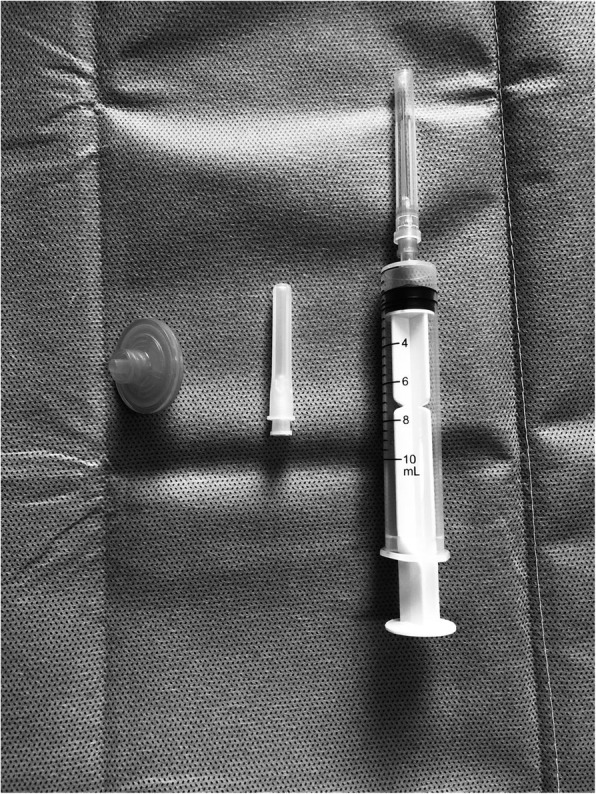
A 10 mL syringe, a 0.22-μm pore size filter and one 30-gauge needle. (Picture Taken by Qun Wang)

Topical anesthesia was administered, and then the conjunctiva and conjunctival sac were fully rinsed with 5% povidone-iodine. The patient was in a seated position. A 10 ml syringe with a 0.22-μm pore size filter was used to inhale 8 ml of sterile air, see Fig. 1. Then a 30-gauge needle was fitted to the 10 ml syringe without the filter, the eye was opened with a speculum and the patient was told to look forward at a fixed target with the other eye. The syringe was inserted into the vitreous at the 6 o’ clock position, 3.5 or 4 mm from the limbus according to the lens status. Before proceeding further, it was confirmed that the needle tip was inserted into the vitreous cavity under direct visualization. While using the left hand to fix the syringe and the right hand to control the needle’s direction, we slowly injected 0.5 ml of gas into the vitreous cavity to increase the intraocular pressure, and gently removed 0.5 ml of vitreous fluid from the eye. It was observed that the liquid containing blood from the vitreous cavity entering through the needle and accumulated in the lower part of the syringe, while the residual 7.5 ml of gas stayed in the upper part of the syringe. 0.5 ml of gas was injected into the vitreous cavity and 0.5 ml of intraocular fluid was elicited. The needle was then slightly withdrawn slowly, so that the needle tip was always kept below the gas-liquid interface in the vitreous. This procedure was repeated until about 4–5 ml vitreous fluid was exchanged into the syringe. At the final step, 0.5 ml of gas was injected into the vitreous to maintain a slightly higher intraocular pressure than normal, then the needle was withdrawn. During the sequential “push pull” cycles, one can evaluate the pressure by gently moving the needle and observing how pliable the globe was. After the procedure, the intraocular pressure was assessed with finger tension.

In the end of the procedure, all patients were given a subconjunctival injection of dexamethasone 2.5 mg in the temporal conjunctiva while in a seated position. Additionally, they were given a topical administration of antibiotic eye ointment 4 times a day for 1 week, and were told to keep a prone position until the air was absorbed to help situate the air in the correct location. After the exchange procedure, additional treatments were given when needed. All cases were followed up for at least 6 months after the last procedure.

### Statistical analysis

The decimal visual acuity was converted to a logarithmic scale for the minimum angle of resolution (LogMAR) used in the statistical analysis. Hand motion visual acuity was converted to 2.28 LogMAR according to a previous investigation [13]. IBM SPSS Statistics 21, (IBM Inc., Armonk, NY, USA), was used for statistical analysis with the level of statistical significance set at *p* < 0.05.

## Results

The details of the age and gender of the patients involved in the study, diagnosis, time of VH occurrence post PPV, numbers of the air-fluid exchange procedures, and any additional treatments are given in Table 1. With the average age of 50.8 ± 12.0 years old, 27 men and 20 women between the ages of 23–77 years old were retrospectively reviewed. Of the 47 patients, 4 patients (8.51%) were diagnosed with T_1_DM, and the 43 patients (91.5) were diagnosed with T_2_DM. Thirty-three eyes (70.2%) developed Early VH post PPV, 13 eye (27.7%) developed delayed VH post PPV, and 1 eye (2.13%) developed severe persistent VH post PPV. The mean follow-up period posts the last exchange procedure was 22.7 ± 13.6 months. The average days of VH post PPV was 34.9 ± 42.7. The original indications for PPV were VH and VH combined with TRD. Forty-four eyes (93.6%) were diagnosis as VH, and 3 eyes (6.38%) were diagnosed as VH plus TRD (less than one papillary disc size).

Of the 47 eyes, 26 of them (55.3%) were phakic, and 21 eyes were pseudophakic. With a careful manipulation during the procedure, no lens injury or injury related cataract formation was found during the follow ups. No cataract surgery was required until the last visit.

The mean number of one-way air-fluid exchange procedures per eye was 1.13. Forty-two eyes (89.4%) needed the air-fluid exchange procedure only once and 4 eyes (8.51%) underwent the procedure twice. One eye (2.13%) was given the air-fluid exchange procedure 3 times. Additional retinal photocoagulation was performed in 41 eyes (87.2%). The patients who was given the procedure three times was diagnosed with T_1_DM and developed severe persistent VH present since postoperative day 1 and nonclearing for 37 days. Five days after the air in the vitreous was absorbed post third exchange procedure, an intravitreal Conbercept was given to prevent recurrent bleeding. Next, a third retinal photocoagulation was followed 7 days later. At the final follow-up, 6 months post final exchange procedure, the fundus was finally stable with no occurrence of any vitreous hemorrhage.

The visual acuity at baseline ranged from hand motion to 20/200. Visual acuity of 4 eyes (8.51%) ranged from hand motion to counting fingers, while that of 43 eyes (91.5%) was worse than 20/200 but better than counting fingers. At 6 m visit, visual acuity of 17 eyes (36.2%) improved to the range between 20/50 and 20/20, and 28 eyes (59.6%) improved to a range between 20/100 and 20/70. Two eyes (4.26%) improved from hand motion to less than 20/200. To sum up, at the final follow-up, the visual acuity was significantly improved in all 47 cases (100%), compared with the visual acuity assessments results at the baseline. See Table 2.

**Table 2:** Snellen Visual acuity outcomes pre and post air-fluid exchange procedure

Visual Acuity	HM or CF	≤20/200	20/100–20/70	20/50–20/20
Pre-procedure	4	43	0	0
6 m Post last procedure	0	2	28	17

*HM* Hand movement, *CF* Counting finger

No major intra-procedure or post-procedure complications were encountered, and air-fluid exchange manipulations were easily performed.

## Discussion

Nonclearing vitreous hemorrhage is the major reason for a post vitrectomy vitreous cavity fluid/fluid exchange or fluid/air exchange operation. The rate of vitreous hemorrhage post PPV has been reported to be between 7 and 63% [1, 2, 14–19], with 9.7% of patients requiring another surgery [12]. Some surgeons may perform PPV when there is rehemorrhage in vitreous of PDR patients post vitrectomy. Our findings showed one-way air fluid exchange is an alternative method to PPV for the treatment. Fluid/fluid exchange methods have the advantage of early visual recovery and various methods of fluid/fluid exchange have been invsestigated [6–11]. Air-fluid exchanges have been reported to be performed on outpatient for vitreous hemorrhage post vitrectomy [20–23], the most widely used method involves fluid/air exchange using a single or two-syringe technique. The technique we used is a one-way method with a single syringe and a 0.22 μm size filter.

In terms of the air-fluid exchange procedure, we suggest the following: 1. Once you have exchange some hemorrhage fluid by injecting air or gas into the vitreous, reinjecting the gas into the air space and taking care to redirect the needle tip into the fluid component to aspirate help make the exchange more effective and easier. 2. The “dead space” in the needle hub can make up for a delay in both steps of the exchange (in and out). Holding the syringe with the needle well above the shaft allows the accumulating bloody vitreous fluid to flow to the back of the syringe and facilitate the air (or gas) being available to enter. 3. Broad fluctuations in the IOP during such a procedure need to be controlled to the minimum with smooth and gentle treatment.

Vitreous cavity hemorrhage following PPV can be present from the first day postoperative and last for a long time (persistent in 20 to 63% of patients), or can occur within the first 4 to 6 weeks (early—5%), or thereafter (delayed—8%) [1, 13, 24]. Early vitreous hemorrhaging is mainly caused by incomplete intraoperative hemostasis, bleeding from dissected fibrovascular tissue or the release of erythrocytes from residual peripheral vitreous gel and iatrogenic injury to the retina or retinal vessels [25]. In our study, 5 cases (10.6%) which were categorized as early VH didn’t need additional treatment post the air-fluid exchange procedure, and the fundus stabilized with no recurrence of VH or retinopathy development. Length of the air-fluid exchange procedure was based on the individual patient’s condition. Forty-one of the 47 patients (87.2%) were given retinal photocoagulation once, post fluid/air exchange procedure. Recurrent vitreous hemorrhage after PPV has been associated with elevated vitreous levels of vascular endothelial growth factor (VEGF). The patient who was given the air fluid exchange procedure 3 times had poor blood glucose control, and the blood glucose control was not stabilized until the application of insulin pump. We evaluated this patient’s condition and recommended anti-VEGF therapy combined with retinal laser. At the final follow-up, his fundus was stable with no recurrence of vitreous hemorrhage. Regarding the efficacy of the one-way air-fluid exchange for a treatment option for vitreous rehemorrhage post vitrectomy on PDR patients, it is important not to neglect the number of eyes requiring repeat air-fluid exchange procedures and maybe PPV was finally referred to a non-clearing hemorrhage. Martin and McCuen had reported that the mean number of air-fluid exchange procedures that every patient was given 1.5, while 40% required another vitrectomy [26]. Han had found out that the mean number of air-fluid exchange procedures every eye was 1.75, while 25% eyes required another vitrectomy [27]. In the study of Behrens AW, the average times of per eye receiving the air-fluid exchange procedures was 1.2 [28]. In our study, the mean number of one-way air-fluid exchange procedures per eye was 1.04, with 41 of 47 eyes (87.2%) requiring additional retinal laser and 1 of 47 eyes (2.13%) requiring intravitreal application of anti-VEGF agent. These results showed that during the first PPV, appropriate and adequate retinal photocoagulation is essential for the post-surgery prognosis of PDR patients.

In our study, there was no significant cataract formation or development post the procedure, while the incidence of post-surgical cataract formation or development have been reported to be 59% of the eyes according to the previous studies [6, 26–28]. In these studies, most of the eyes are phakic, while in our study, 26 eyes (55.3%) were phakic. Additionally, other complications such as iris neovascularization and anterior hyaloidal fibrovascular proliferation, glaucoma had been reported [6, 26–28]. In our study, other than the air-fluid exchange procedure, additional treatments were given respectively provide an explanation for the low incidence of the above complications. Compared with the vitrectomy in the post era, the development of PPV, surgical techniques and managements discourage the formation of the fibrosis and alleviation of the retinal ischemia [29, 30].

A 10 ml syringe with a 0.22-μm pore size filter was used to inhale sterile air. The 0.22-μm pore size filter can be combined with the vitrectomy machine cannula and help to reduce the incidence of intraocular infection, which was not reported before that it could be equipped with a syringe. This technique was employed on 47 eyes. A clear fundus was achieved the moment the air was absorbed, and no infectious complications being observed during the follow-ups. This approach produced consistent repeatable results.

This technique also has the following advantages. Firstly, due to its simplicity, surgeons can perform the procedure without assistance and a long learning curve is not needed. Secondly, it enables surgeons to make more informed or accurately decisions whether full reoperation is necessary. Lastly, it is minimally invasive and topical anesthesia is adequate for this procedure, as this is a one way entering vitreous procedure. The 30-gauge sclerotomy did not leak with non-beveled insertion, and was self-sealing, however it does have a disadvantage of causing the intraocular pressure to fluctuate during the procedure.

This study also has some limitations as it is a small size, retrospective design study with short follow-up time, which may owe to selection bias. Because that cases with more severe vitreous hemorrhage may have already been treated with a second vitrectomy. This may explain the small sample size of our study. Despite these limitations, we believe that our result demonstrate the use of one-way air-fluid exchange in the setting of vitreous rehemorrhage post vitrectomy for PDR patients.

## Conclusion

This one-way air-fluid exchange procedure can effectively exchange the vitreous hemorrhage and improve visual acuity without obvious complications on PDR patients who develop rehemorrhage post vitrectomy.

## Data Availability

No Identifying/confidential patient data should not be shared in public. If anyone who is interested in the primary data of the patients could contact the corresponding authors for permission of the data access.

## Abbreviations

PDR: Proliferative diabetic retinopathy
BCVA: Best corrected visual acuity
T1DM: Type 1 diabetes mellitus
T2DM: Type 2 diabetes mellitus
PPV: Pars plana vitrectomy
VH: Vitreous hemorrhage
LogMAR: Logarithmic scale for the minimum angle of resolution
VEGF: Vascular endothelial growth factor

## References

[1] Khuthaila MK, Jason H, Allen C, Char DCF, Milder Eugene A, Vikram S, Garg Sunir J, Spirn Marc J (2013). Postoperative vitreous hemorrhage after diabetic 23-gauge pars plana vitrectomy. Am J Ophthalmol.

[2] Yorston D, Wickham L, Benson S, Bunce C, Sheard R, Charteris D (2008). Predictive clinical features and outcomes of vitrectomy for proliferative diabetic retinopathy. Br J Ophthalmol.

[3] Enrique S-P, Concepcion H-OM, Antonio VJ (2005). Risk factors for postoperative hemorrhage after vitrectomy for diabetic retinopathy. Ophthalmic Epidemiol.

[4] Saori M, Nobuhiko S, Takuma I, Hirokazu S, Daijiro K, Mitsuyoshi T, Takekazu K, Junji K, Akihisa I, Kohji N, Ayumi S, Hiromi I, Iichiro S (2018). Predictors of postoperative bleeding after vitrectomy for vitreous hemorrhage in patients with diabetic retinopathy. J Diabetes Investig.

[5] Yoshihiro W, Yoshihiko U, Kinya T, Shunichiro U, Kazuhiko U, Daisuke M, Hiroshi G (2017). Persistent overproduction of intraocular vascular endothelial growth factor as a cause of late vitreous hemorrhage after vitrectomy for proliferative diabetic retinopathy. Retina.

[6] Blankenship GW (1986). Management of vitreous cavity hemorrhage following pars plana vitrectomy for diabetic retinopathy. Ophthalmology.

[7] Miller JA, Chandra SR, Stevens TS (1986). A modified technique for performing outpatient fluid-air exchange following vitrectomy surgery. Am J Ophthalmol.

[8] Saito Y, Emi K, Danjo S, Tanaka F (1992). Simplified vitreous lavage for bleeding after vitrectomy. Ophthalmic Surg.

[9] Eter N, Spitznas M (2002). A new and simple method for performing vitreous lavage. Retina.

[10] Wu WC, Chen JY, Chen YC, Chang YC (2009). Management of post vitrectomy diabetic vitreous hemorrhage with volume homeostatic fluid-fluid exchanger. Graefes Arch Clin Exp Ophthalmol.

[11] Dongqing Z, Jing Z, Jibo Z (2018). One-port vitreous cavity lavage with hybrid 27G infusion and 23G cannula. Eur J Ophthalmol.

[12] Gupta B, Sivaprasad S, Wong R, Laidlaw A, Jackson TL, McHugh D, Williamson TH (2012). Visual and anatomical outcomes following vitrectomy for complications of diabetic retinopathy: the DRIVE UK study. Eye.

[13] Holladay JT (1997). Proper method for calculating average visual acuity. J Refract Surg.

[14] Lee BJ, Yu HG (2010). Vitreous hemorrhage after the 25-gauge transconjunctival sutureless vitrectomy for proliferative diabetic retinopathy. Retina.

[15] Cheema RA, Mushtaq J, Cheema MA (2010). Role of residual vitreous cortex removal in prevention of postoperative vitreous hemorrhage in diabetic vitrectomy. Int Ophthalmol.

[16] A Kamura Y, Sato Y, Deguchi Y, Yagi F (2013) Iatrogenic retinal breaks during 20-gauge vitrectomy for proliferative diabetic retinopathy. Clin Ophthalmol 7:29–33. 10.2147/OPTH.S38784. 10.2147/OPTH.S38784 PMC3534296 23293512

[17] Landers MB, Perraki AD (2003). Management of post-vitrectomy persistent vitreous hemorrhage in pseudophakic eyes. Am J Ophthalmol.

[18] Hershberger VS, Augsburger JJ, Hutchins RK, Raymond LA, Krug S (2004). Fibrovascular ingrowth at sclerotomy sites in vitrectomized diabetic eyes with recurrent vitreous hemorrhage: ultrasound biomicroscopy findings. Ophthalmology.

[19] Yeh PT, Yang CM, Yang CH, Huang JS (2005). Cryotherapy of the anterior retina and sclerotomy sites in diabetic vitrectomy to prevent recurrent vitreous hemorrhage: an ultrasound biomicroscopy study. Ophthalmology.

[20] Sofia P, Fine Howard F (2018). A novel approach to in-office air-fluid exchange utilizing a 27-gauge Valved trocar cannula. Ophthalmic Surg Lasers Imaging Retina.

[21] Xie ZG, Chen F, Tong J, Zhu J, Du W (2016). Two-way fluid-gas exchange syringe for Postvitrectomy patients in outpatient setting. Retina.

[22] Mason RW (2014). Single incision, outpatient fluid/gas exchange. Retina.

[23] Kwok AK, Lam DS, Cheng LL, Sharma T (1998). Outpatient postoperative fluid-gas exchange after early failed vitrectomy surgery. Ophthalmology.

[24] Novak MA, Rice TA, Michels RG, Auer C (1984). Vitreous hemorrhage after vitrectomy for diabetic retinopathy. Ophthalmology.

[25] El Annan J, Carvounis PE (2014). Current management of vitreous hemorrhage due to proliferative diabetic retinopathy. Int Ophthalmol Clin.

[26] Martin DF, McCuen BW 2nd (1992). Efficacy of fluid-air exchange for postvitrectomy diabetic vitreous hemorrhage. Am J Ophthalmol.

[27] Han DP, Murphy ML, Mieler WF, Abrams GW (1991). Outpatient fluid-air exchange for severe postvitrectomy diabetic vitreous hemorrhage long-term results and complications. Retina.

[28] Behrens AW, Uwaydat SH, Hardin JS, Sallam AB (2019). Office-based air-fluid exchange for diabetic post-operative vitreous cavity hemorrhage. Med Hypothesis Discov Innov Ophthalmol.

[29] Iglicki M, Zur D, Busch C. Results in comparison between 30 gauge ultrathin wall and 27 gauge needle in sutureless intraocular lens fanged technique in diabetic patients - 24 months follow up study. Acta Diabetol. 2020;57(10). 10.1007/s00592-020-01530-8. 10.1007/s00592-020-01530-8 32300875

[30] Matias I, Dinah Z, Adrian F, Pierre-Henry G, Marco L, Rodrigo S, Catharina B, Matus R, Zafer C, Martin C, Dua M, Shulamit S, Adiel B, Anat L, International Retina Group (IRG) (2019). TRActional DIabetic reTInal detachment surgery with co-adjuvant intravitreal dexamethasONe implant: the TRADITION STUDY. Acta Diabetol.

